# Vegetation cover and seasonality as indicators for selection of forage resources by local agro-pastoralists in the Brazilian semiarid region

**DOI:** 10.1038/s41598-022-18282-w

**Published:** 2022-09-07

**Authors:** Sonaly Silva da Cunha, Maiara Bezerra Ramos, Humberto Araújo de Almeida, Maria Gracielle Rodrigues Maciel, Stefanny Martins de Souza, Kamila Marques Pedrosa, Sérgio de Faria Lopes

**Affiliations:** 1 Laboratório de Ecologia Neotropical, Departamento de Biologia, Centro de Ciências Biológicas e da Saúde, Universidade Estadual da Paraíba, Bairro Universitário, Campina Grande, Paraíba 58429-500 Brazil; 2 Laboratório de Fisiologia Vegetal, Departamento de Botânica, Universidade Federal de Minas Gerais, Av. Antônio Carlos, 31, Belo Horizonte, Minas Gerais 270-901 Brazil

**Keywords:** Biodiversity, Community ecology, Grassland ecology, Sustainability

## Abstract

Local knowledge and uses of forage resources are highly dynamic, and can be mediated by multiple factors, such as seasonality, floristic diversity and the morphophysiological characteristics of plants. We investigate how seasonality and vegetation cover mediate the use of forage resources. The study was carried out with agro-pastoralists from two areas of Brazilian semiarid region. To select the areas, we used the normalized difference vegetation index. We selected one area with low vegetation cover (Area I) and another with high vegetation cover (Area II). Respondents were selected using the snowball technique. Using semi-structured interviews, we collect the information about forage use in the dry and rainy seasons, preferences of ruminants and specific characteristics of plant species. A total of 57 informants were interviewed in the two areas. We used the Chi-square test to assess differences in the richness of species cited between areas, seasons (dry/rainy), origins (exotic/native) and strate (herbaceous/woody). Our results revealed that agro-pastoralists living in the area with the highest vegetation cover (Area II) cited a greater number of species. We found that the use and selection of species is guided by a series of functional characters, related to palatability and nutritional value, which change between seasons. These results highlight the vast knowledge of ecological characteristics of species and diet of ruminants acquired by agro-pastoralists during field experience, with seasonality defining the use of species. Different from what we expected, the richness of exotic species mentioned did not differ between areas. This diversity of information contributes to a better understanding of the use of forage resources and indicates the importance of including local experiences as strategies to ensure proper use and provide insights for the conservation of local resources.

## Introduction

Plant biodiversity offers a large number of ecosystem services that have historically been exploited by human populations^1^. Such interactions have led to the accumulation of a triad of knowledge, belief and practice by people^2^ that is mediated by a diversity of social and ecological factors^3^. From an ecological point of view, environments are highly heterogeneous, and thus the supply of resources is irregular and varies not only in relation to availability but also in quality^4^. Considering that populations maintain intrinsic relationships with these resources, the effects of such variation can shape their usage profile according to the environmental context in which they are inserted, resulting in different usage patterns and extensions of local knowledge^5,6^.

The supply of resources and the floristic diversity of an environment is positively correlated with the degree of knowledge and use^5–7^. Indeed, ethnobotanical works have shown a greater citation of species by informants inserted in places with greater vegetation coverage and environmental heterogeneity^6^. This diversity of species not only influences citation richness but offers an opportunity to choose the use of resources and the use of specific species^5^. On the other hand, environments with less resources, as a result of anthropogenic actions, may favor less specialized patterns of use due to limited availability of species^8^. An adaptive strategy for this scarcity of resources is to increase use of exotic species to meet demands not satisfactorily fulfilled by native species^9^.

The Brazilian Caatinga, a dry forest of tropical domain, is the target of multiple exploitations of plant resources, such as for timber, energy, medicinal and forage purposes, the uses of which are particularly modulated by a characteristic pattern of seasonality^10^. In addition to this idiosyncratic factor, the irregularity of rainfall in the region has also transformed socioecological systems to the extent that livestock has contributed substantially to the economy in parallel with agriculture activities^11^, with goats and sheep composing the main types of livestock that are kept free-grazing on native vegetation^12^.

Although historically consolidated, maintaining livestock remains a challenge for producers, especially during the dry season when the supply of fodder becomes limited^11^. As a result, producers are negatively affected and generally must reduce herds during this period to maximize use for a better return, which in turn contributes decisively to the animal foraging patterns^13^. Management of these domestic animals has provided a vast knowledge of the potential of forage species and has concomitantly contributed to the aggregation of different criteria for characterizing of forage resources^14–17^. Breeders are able to discern which species are consumed by animals in different seasons, as well as animal preferences for consumption^16^, and consequently assess the quality of forage based on its nutritional properties^14,16^.

Understanding such foraging dynamics can regulate local preferences of populations for certain species, which can be given by the content of nitrogen and dry matter, for example^14^. However, identifying the qualitative character used in people’s perceptions is sometimes complex, since less explicit forms of associating quality of forage with the effect on the animal can be found, such as weight gain or increased milk production^17^. In this context, and considering the aforementioned ecological perspective involving the use and knowledge of forage resources, we hypothesized that there is a positive relationship between the richness of cited forage species and areas with greater vegetation coverage. We also believe that environments with less vegetation cover will present a greater richness of cited exotic forage species since native resources are less abundant. We also sought to understand the dynamics of forage resources, including the use of exotic species and plant strata in view of seasonal characteristics and vegetation cover.

## Materials and methods

### Study area within a broad regional context

**Figure 1:**
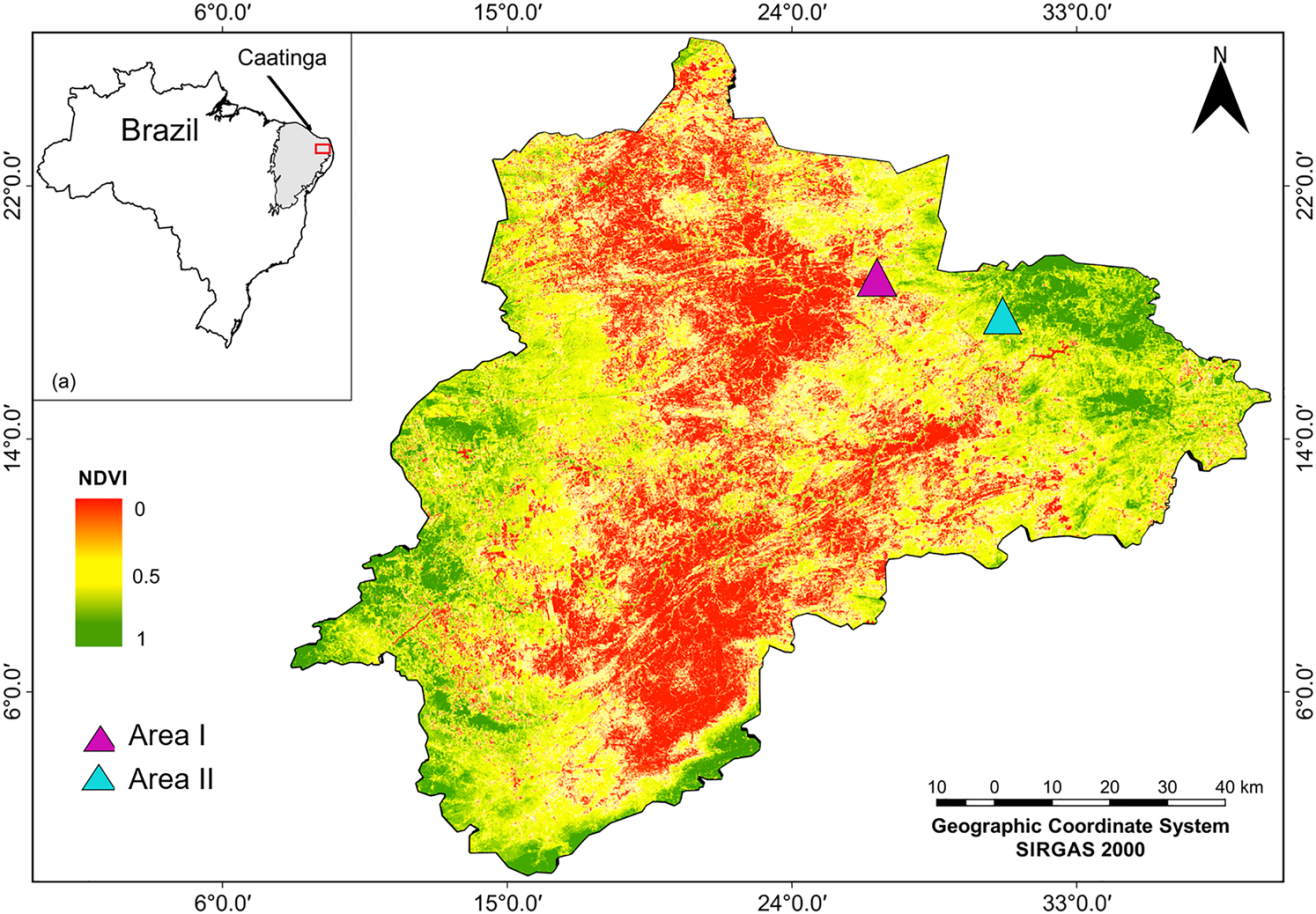
Map of vegetation cover for the Cariri region, Paraíba state, in the Caatinga realm, and its location in northeastern Brazil (left upper inset). The purple triangle indicates the area with the lowest vegetation coverage (Area I, municipality of São João do Cariri). The blue triangle indicates the area with the highest vegetation coverage (Area II, Municipio de Cabaceiras). The vegetation cover map was elaborated from the vegetation index by normalized difference NDVI. The map created with QGIS 2.18.0 (https://qgis.org/en/site/).

The study was carried in the Cariri microregion in the state of Paraíba, in the semi-arid region of Brazil (Fig. 1). The region of Cariri has a total area of approximately 11,225.736 km^2^ and a particular landscape diversity. Regional climate is hot, semi-arid (Bswh type)^18^. Marked by low rainfall regimes with annual precipitation between 350 and 700 mm and high variability in space and time. The mean annual temperature is 26 °C, with minimum values in July and August and maximum values in November and December^19^. The altitude varies from 250 m above sea level (m.a.s.l.) in valleys to 1,180 m.a..s.l. in the cliffs and inselbergs^20^.

The dominant vegetation type is the seasonally dry tropical forest, known in the country as Caatinga. Its flora encompasses numerous species having morpho-physiological adaptations in response to chronic water stress^21,22^. Relatively short trees dominate the Caatinga, most with heights ca. 10 m, along with numerous species of shrubs, cacti, and bromeliads^22^.

The extensive raising of herds, mainly goats, sheep and cattle, where the animals graze freely through extensive areas of native vegetation, is considered one of main economic activities in the region, and reflects a historical relationship. Started around sixteenth century, as the European conquest advanced in Brazil’s semi-arid region land donations through sesmaria letters promoted the settlement of these regions and the expansion, agricultural production, and allocate cattle in drier regions^23,24^.

### Selection of studied communities and informants

To test our hypothesis that vegetation cover would influence the richness of the cited forage species, we sought to select an area with low vegetation cover and an area with high vegetation cover. In this way, we selected two rural populations that develop activities agricultural and livestock activities; agro-pastoralists living an area with low vegetation coverage and agro-pastoralists living an area with greater vegetation coverage, in the municipalities of (São João do Cariri, (36° 27′ 6.543″ W, 7° 23′ 47.424″ S) and Cabaceiras (36° 15′ 4.50″ W, 7° 26′ 24.90″ S), respectively. Both populations are located in the Cariri microregion in the state of Paraíba, in the semi-arid region of Brazil (Fig. 1). The areas were selected according to different levels of vegetation cover given by the normalized difference vegetation index (NDVI), which can serve as a proxy to estimate biomass and vegetation productivity by calculating NDVI = (Infrared − Red)/(Infrared + Red)^25^, given by ArcGIS Software. Data for NDVI were obtained from images of bands 4 and 5 of Landsat8 satellite, referring to the dry season, October of the year 2017 and obtained from eartherexplorer.usgs.gov. The bands correspond to the proportion of light reflected by the vegetation and detected by sensors, being designated red and near infrared, respectively. Residences that were within a radius of 5 km of the defined areas were included. Thus, throughout the work the area with the lower vegetation cover (lowest NDVI value, *São João do Cariri*) will be referred to as Area I while the area with the higher vegetation cover (highest NDVI value; *Cabaceiras)* will be referred to as Area II.

### Ethnobiological data collection

The areas were visited prior to conducting interviews in order to establish a relationship of trust with the interviewees, to explain the objectives of the research, and to collect preliminary information from informal conversations. We conducted individual semi-structure interviews during May and June 2019, with questions regarding forage uses corresponding to dry and rainy seasons, preference criteria and ecological characteristics of forage species.

Questions were directed to agro-pastoralists in each area who were selected using the snowball method—the first informants were asked to indicate others, and so on, in succession^26^. The research included those who accepted to be part of the interviews, lived close to the vegetation cover estimated by NDVI and who had experience with raising ruminants.

The interviews were carried out in the homes and sometimes during field work, which allowed the recognition of several species mentioned by the agro-pastoralists. During this moment, direct observations complementary to the interviews were made with regard to herding activities. As plants were identified, photographs were taken and inserted in an album used for later ratification of the cited species and as visual stimulus.

Whenever possible, we collected plant material from the species mentioned for further taxonomic identification. The identifications were confirmed specialist in plant taxonomy and herbarium curator at the State University of Paraíba, *Herbario Manoel Arruda Câmara*—ACAM, where the samples were deposited (see voucher number in Appendix 1).

A total of 57 informants were obtained, 27 for Area I (*São João do Cariri*) and 30 for Area II (*Cabaceiras*). The informants of Area I were represented by 52% women, 48% men, famers, mean age of 58 years and 96% wage earners. Schooling ranged from complete elementary and middle school (45%), incomplete elementary and middle school (15%), complete high school (18%), literate (18%) and never attended school (4%). The informants of Area II were represented by 66% men, 34% women, agro-pastoralists, mean age of 55 years, 80% wage earners, 10% with little more than a minimum wage and another 10% with less than one minimum wage. Schooling ranged from incomplete elementary and middle school (37%), complete elementary and middle school (10%), literate (17%), never attended school (23%), incomplete high school (10%) and complete high school (3%).

Most of the agro-pastoralists plant only in the rainy season. However, as a result of frequent droughts, these activities have been less frequent, with extensive breeding of goats, sheep and, to a lesser extent, cattle, being the main source of contribution to income.

### Ethical aspects

All methods were carried out in accordance with relevant guidelines and regulations. The study was submitted and approved by the Ethics and Research Committee of *Centro Universitário Unifacisa*, opinion n^o^: 010713/2019 and Ethical Certificate number (07564918.2.0000.5175). We conducted surveys and interviews to agro-pastoralists after obtaining prior informed consent from each one of them. Only those who agreed to participate and sign the form were included in the research, in accordance resolution 510/2016 of *Conselho Nacional de Saúde*. The collection of plant material was permission by the *Sistema de Autorização e Informação em Biodiversidade do Brasil*—Sisbio, under number 68447.

### Data analysis

The cited species were classified according to strata and biogeographic origin based on the literature and the website *Flora do Brasil*^27^. For the strata category the species were classified as woody or herbaceous, while for the biogeographic origin category the species were classified as exotic or native. The uses of the species cited by the agro-pastoralists were also organized according dry or rainy season. In all analyses, comparisons were made between areas and seasons considering only species that have been scientifically identified.

Variables were tested for normality by the Shapiro test; however, all *p*-values were < 0.05. Differences in the richness of species cited between areas, seasons (dry/rainy), origins (exotic/native) and strate (herbaceous/woody) were evaluated using the Chi-square test. Chi-square was also used to test differences in origins and strata of the cited species between seasons.

Composition of species cited in the two areas was evaluated by Non-metric Multidimensional Scaling (NMDS) using the Bray–Curtis similarity index. Ordering was carried out using a matrix with the code of the interviewees and the abundance data of forage species cited in the seasons. As this test is a parametric analysis, data were transformed into log of x + 1 to adjust the distribution.

The significance of the difference between the formed groups was obtained by Permanova and similarity through clustering using the Morista index. Tests of normality, Chi-square, NMDS and Permanova were performed using the program R version 3.2.2^28^.

## Results

### Characterization of forage species

A total of 70 species distributed among 26 families were cited for Area I. The families with the greatest floristic richness were Fabaceae (12 species cited), Poaceae (10), Cactaceae (6) and Malvaceae (4) (Appendix 1). The cited species included 45 natives and 17 exotics, and 32 woody and 30 herbaceous. While in Area II, a total of 80 species distributed among 29 families were cited, with the most represented families being Fabaceae (14), Poaceae (8), Malvaceae (7), Euphorbiaceae (6) and Cactaceae (6) (Appendix 1).

In both areas, the rainy season had a greater number of citations for herbaceous species and a greater presence of exotic and unique species compared to the dry season, while the dry season had a higher number of woody species and fewer exotics. The species included 55 natives and 16 exotics, with 38 arboreal and 34 herbaceous (Table 1).

**Table 1:** General characterization of the parameters of the species mentioned in areas of Caatinga with different levels of vegetation cover, low coverage (Area I) and high coverage (Area II), semi-arid region of Brazil.

Parameters	Area I			Area II		
	Rainy	Dry	Total	Rainy	Dry	Total
Family	24	19	26	29	19	29
Richness	48	41	70	67	44	80
Unique species	21	14	35	29	5	34
Woody	23	30	32	33	36	38
Herbaceous	25	11	30	34	8	34
Native	33	32	45	52	37	55
Exotic	15	9	17	14	7	16

**Figure 2:**
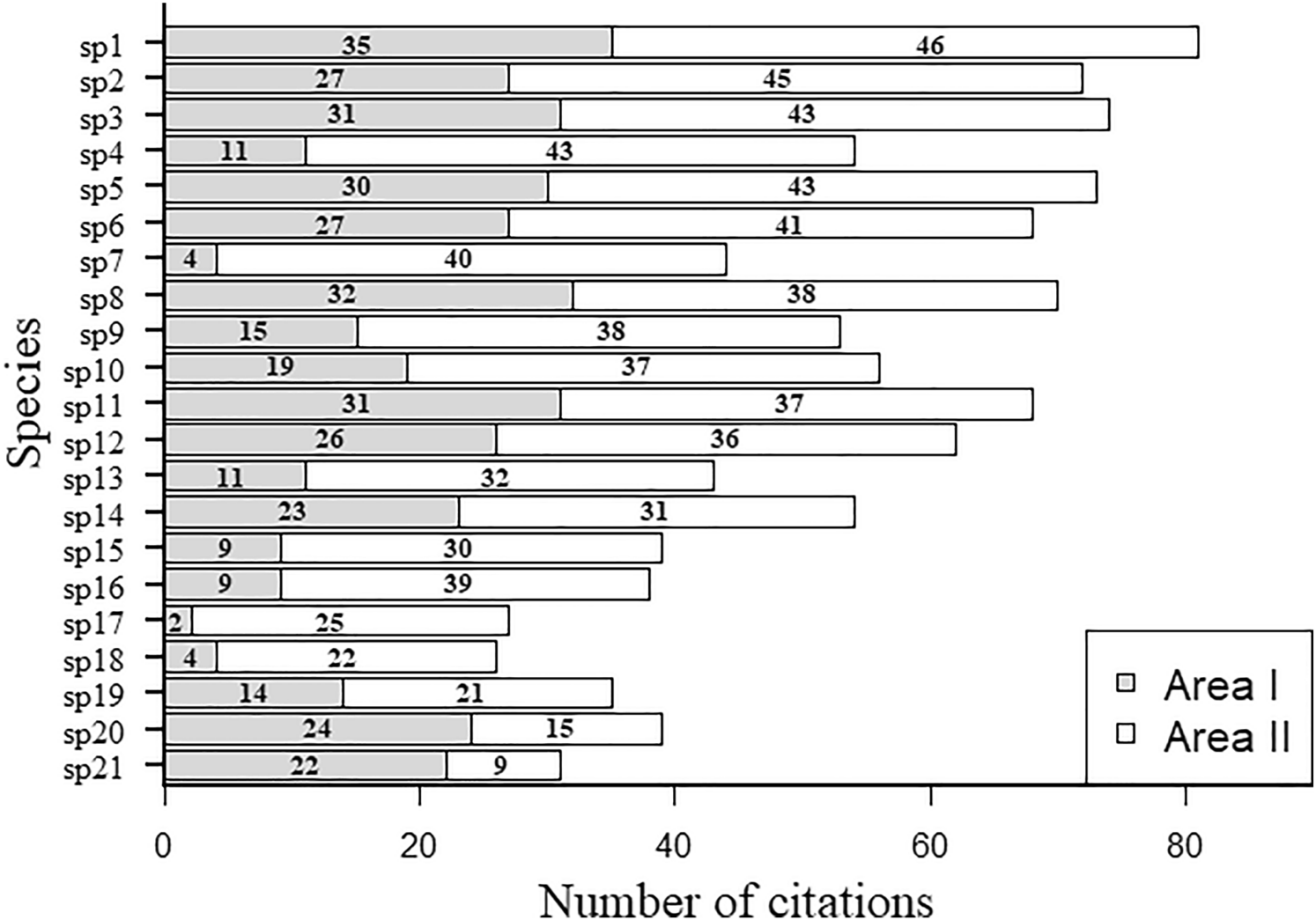
Forage species most cited by local specialists in Caatinga areas with low (Area I) and high vegetation cover (Area II), located in the municipalities of São João do Cariri and Cabaceiras, respectively, of vegetation cover semi-arid region of Brazil. sp1 (*Cenostigma pyramidale*), sp2 (*Spondias tuberosa*), sp3 (*Croton Blanctenianus*), sp4 (*Manihot glaziovi*), sp5 (*Ziziphus joazeiro* ), sp6 (*Myracrodruon urundeuva*), sp7 (*Mimosa tenuiflora*), sp8 (*Sideroxylon obtusifolium*), sp9 (*Pilosocereus pachycladus*), sp10 (*Cynophalla flexuosa*), sp11 (*Aspidosperma pyrifolium*), sp12 (*Pilosocereus gounellei),* sp13 (*Melocactus zehntneri*), sp14 (*Bromelia laciniosa*), sp15 (*Commiphora leptophloeos*), sp16 (*Anadenanthera colubrina*), sp17 (*Piptadenia stipulacea*), sp18 (*Bauhinia cheilantha*), sp19 (*Mimosa ophthalmocentra*), sp 20 (*Prosopis juliflora*), sp21 (*Tabebuia aurea*).

Ten species stood out as highly cited for Area I, while 20 species stood out as highly cited for Area II (Fig. 2). In general, native species, regardless of the season, were cited more than exotic species in both areas, however, at a considerably higher proportion in Area I (low coverage) (Table 1). With the exception of the exotic species *Prosopis juliflora*, which most cited for Area I, and native *Tabebuia aurea,* the other most cited species were also present in Area II (Appendix 1).

### Climatic seasonality and forage species

**Figure 3:**
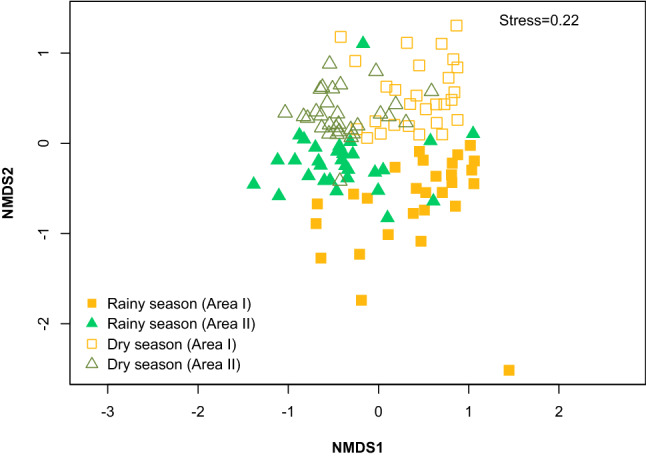
Non-metric multidimensional scaling ordination (NMDs), based on the Bray–curtis index, grouping between dry and rainy seasons in Caatinga areas with different levels of vegetation cover semi-arid region of Brazil (two-dimensional solution, stress = 0.22) Triangles and filled squares show species from the rainy season (Area I and Area II, respectively) and triangles and empty squares from the dry season (Area I and Area II, respectively).

The ecological parameters of the mentioned species differed between the areas. Area II had greater richness of plants cited (X^2^ = 2251.7; *p* < 0.05), and a predominance of woody (X^2^ = 156.75; *p* < 0.05) and native (X^2^ = 173.19; *p* < 0.05) species than Area I. However, there was no significant difference between areas for herbaceous habit and exotic species. On the other hand, there was a difference between seasons for each area in the composition of species cited (F = 14.974; *p* < 0.05). The NMDS formed two clusters with overlapping patterns (Fig. 3), one group comprising the rainy seasons and the other the dry seasons. Similarity was greater between dry seasons (87% similarity) than between rainy seasons (81% similarity).

With regard to stratum, significant differences were observed between areas the for herbaceous species (X^2^ = 0.1380; *p* < 0.05) cited in the rainy seasons, but not for herbaceous species cited in the dry season. For woody species, Area II had a greater richness of species cited for the rainy (X^2^ = 100.23; *p* < 0.05) and dry (X^2^ = 109.57; *p* < 0.05) seasons.

It was also found that agro-pastoralists in Area II cited more native species (X^2^ = 105.06; *p* < 0.05) in the rainy season and in the summer (X^2^ = 116.73; *p* < 0.05). However, no difference was observed between areas in the use of exotic species in either season.

### Ecological knowledge about forage species

**Figure 4:**
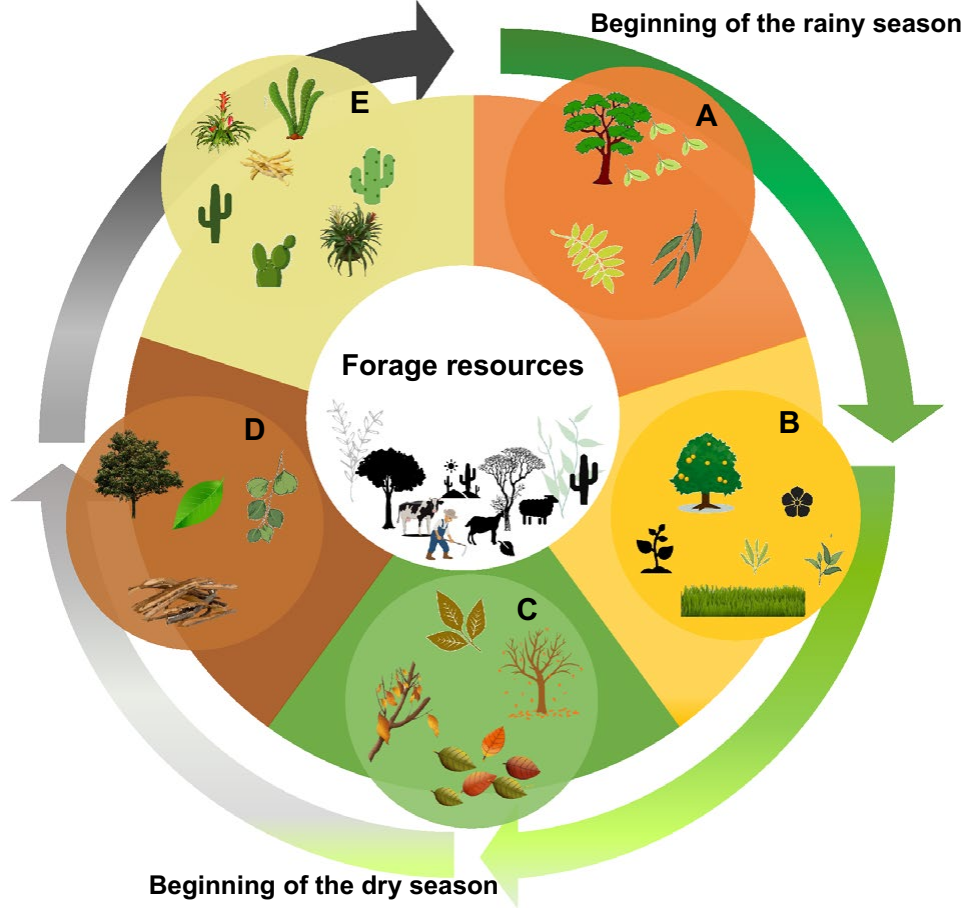
Diagrammatic representation for the knowledge of plants used as forage in the Brazilian semi-arid region. The central, blank panel is the representation of a local expert in the field. The arrows illustrate in an illustrative way the gradient of vegetation cover. The arrow starts in dark green, which marks the beginning of the rainy season, and ends in dark gray, which marks the peak of the dry season. The circles represent the five groups of plants preferred by the herds according to quotes from local experts. Orange circle (**A**), here are the young leaves of the woody species. Yellow circle (**B**) herbaceous, grasses, fruits and leaves of woody species. Green circle (**C**) Ripe and dry leaves. Brown circle (**D**) woody bark, and leaves of evergreen species. Beige circle (**E**) Cactaceae and Bromeliaceae. Image created with Microsoft Office 2019 PowerPoint (www.office.com).

The agro-pastoralists exhibited extensive knowledge about forage species and were able to distinguish animal consumption between and within seasons, as well as preferences and phenology, palatability and availability. Figure 4 provides a foraging profile that was drawn according to the information provided by the agro-pastoralists.

The agro-pastoralists characterized different foraging patterns throughout the seasons based mainly on availability, palatability and nutritional value. According to the agro-pastoralists, as soon as the first rains occur the animals consume newly sprouted leaves and have a great preference for softer, more nutritious and non-bitter forage. The species for which leaves were consumed especially at the beginning of rainy season were *Cenostigma pyramidale, Croton blanchetianus* and *Aspidosperma pyrifolium*, thus explaining the high number of citations for these species. However, the agro-pastoralists explained that leaf palatability changes during maturation. The leaves of *C. blanchetianus*, for example, become thicker, while those of *A. pyrifolium* become bitter, which is reflected in changes animal selectivity.

With rains becoming more frequent, herbaceous plants emerge and are preferably consumed because they are more available. Despite not knowing how to identify many of the forage species, the interviewees affirm that grazing activities allow them to make these inferences. Many species also bear fruit during this period, with the fruits of *Spondias tuberosa* and *Ziziphus joazeiro* being highly appreciated, especially those of the former. Seedlings are also highly desired for reasons of greater accessibility and flavor, particularly those of *Myracrodruon urundeuva*.

The beginning of dry season brings greater use of woody species, and mature or dry leaves are consumed because they are available. Furthermore, species that present poisonous or bitter character lose such properties at this time, such as *A. pyrifolium*. If drought continues, agro-pastoralists use cacti and bromeliads, such as *Pilosocereus gounellei, P. pachycladus* and *Bromelia laciniosa*, to complement the diet of ruminants. With the lowest supply of resources, the bark of some species, such as *Myracrodruon urundeuva*, *Spondias tuberosa*, *Croton blanctenianus*, *Amburana cearensis* and *Prosopis juliflora*, among others, is also consumed.

The species consumed throughout the rainy season also influence the reproductive period of ruminants. According to informants, as soon as the first rains occur and forage species become leafy, which they characterize as “ponta de rama” (tip of branch), the animals show greater sexual receptivity because there is a supply of resources that allow them to “encher a barriga” (fill their bellies), become strong and be signaled that keeping offspring is feasible. The interviewees also add that when animals in the reproductive process are confined, they can be fed branches of species that they are known to prefer.

## Discussion

**Figure 5:**
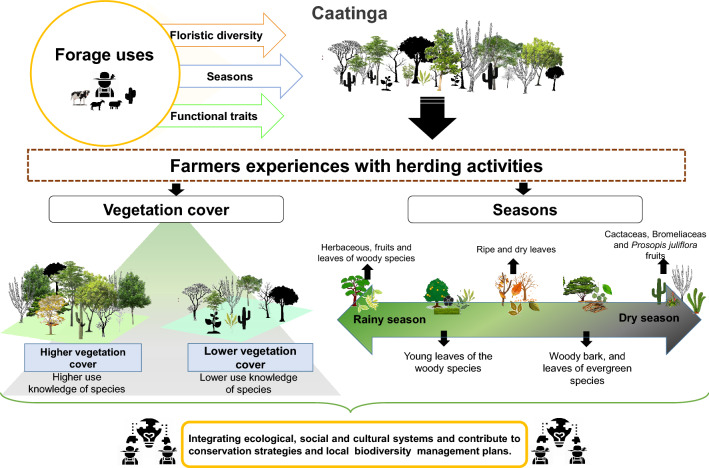
Diagrammatic representation for the effects of vegetation cover and seasonality on forage resource selection in Dry Forests. Image created with Microsoft Office 2019 PowerPoint (www.office.com).

In line with the results of present study, we suggest that the exploitation of forage resources by agro-pastoralists occurs in a non-random manner. The use of forage resources is guided by a series of functional characters related to palatability and nutritional value, which determine preferential use due to the better quality of resource. At the same time, we understand that forage uses are complex and multifactorial in nature, and regulated in a substantial way by seasonality and ecological factors (Fig. 5), such as the availability of plant resources and local diversity.

The differences of plant species cited between areas reveal the positive effect of vegetation cover on the use and knowledge of plants by agro-pastoralists. Our findings reveal that the greater number of plant species mentioned by agro-pastoralists in Area II is directly associated with greater availability of resources in this area, as long as we consider vegetation cover as availability of resources, which allows different species to be used throughout the year. On the other hand, in regions with low vegetation cover (Area I), the low availability of resources limits the use and knowledge of plants by residents, which can lead to greater pressure on a small set of available species. Such findings reinforce the importance of vegetation cover for ecosystem provision of goods and services to human populations that depend directly or indirectly on these services.

The most represented families found in the present study have also been reported in several other ethnobotanical studies^6,16,17,29^, with emphasis on Fabaceae and Poaceae, which are recognized for their high forage potential, which derives, above all, from high palatability and nutritional value^30^. Simultaneously, citations mostly for native species reflect the importance and potential of Caatinga resources as important components of the ruminant diet^11^, both for the woody and herbaceous strata, corroborating the estimate in the literature that 70% of vegetation has potential use as forage^31^.

The characteristic seasonality of vegetation, on the other hand, represents a limiting factor for forage productivity, culminating in high fluctuations in quality and availability, as well as changes in the dominance of different strata and composition of forage species throughout the seasons^11,32^. The seasonal distribution of species explains the similarity of seasons between areas, with a higher similarity percentage for the dry seasons, since there is less availability of resources to be exploited compared to the rainy season. In this context, the potentially used species are commonly accessible woody species in both areas. However, during the rainy season, the high availability of herbaceous plants regulates different uses (Fig. 4), but even so, they also exhibit relatively similar patterns, mainly due to the woody component that denotes the common demand by ruminants at the beginning of this season.

The effect of climatic variables on vegetation use patterns was documented by^16,17^, both of which showed greater richness in the use of herbaceous forage during the rainy season, a finding that reflects the seasonal distribution—restriction to that season—and decrease in the qualitative character of annual species^33^. At the same time, it also reflects the greater number of unique species for the rainy season. However, when compared to woody strata, significant differences in terms of richness are not found because although the diversity of herbaceous species in the Caatinga is greater^24^, it is much less known than that of the tree-shrub stratum^11^.

Agro-pastoralists even characterize animal preferences for herbaceous stratum, but as its diversity is immense and ephemeral, they claim to have limited ability to identify the species. The high abundance of resources in the rainy season also reduces the concern with forage use, which implies less attention to the species that are consumed. In contrast, woody species, due to multiple uses and greater availability over time, tend to be better known^10,34^, with a different effect in the dry season making the optimal foraging pattern in this period inherent to the knowledge of agro-pastoralists^35^.

In addition, according to the ecological appearance hypothesis, there is a general tendency for less apparent species to be neglected by populations^36^. Some studies have corroborated the hypothesis within the context of forage use, with woody species being cited more and having more uses^6,15^. In addition, people tend to focus on resources whose supply is given continuously^10^, which may explain why woody species are well represented in both seasons.

Security in the provisioning of ecosystem services is an essential component for local populations, and thus woody species are highly valued because they reflect predictability of use^15,35^. This can be a particularly influential criterion because perennial or late leaf deciduous species, such as *Cynophalla flexuosa* and *Myracrodruon urundeva*, had significant amounts of citations and perceptions employing high valuation, as represented by some statements by some interviewees: “*É um refrigero na seca*” (it is savage in the dry season), “*É uma ração boa na seca*” (it is a good food in the dry season).

In turn, differences in richness of the species cited by the two areas corroborate our first hypothesis that populations inserted in environments with greater vegetation cover tend to cite more species. In line with these findings, considerable floristic dissimilarity was also found between the two areas, given the exclusivity of species. Such dissimilarity may suggest particularities in the vegetation attributes of each area, such as greater floristic diversity^7,37,38^.

Since anthropic processes are irregularly distributed in space, variation in the provisioning of ecosystem services by vegetation also occurs, and influences different collection profiles^39^. On the other hand, areas with greater species richness have been shown to have greater use patterns^6,7^. The larger number of species cited as woody and native for Area II is, therefore, associated with greater general richness, as well as herbaceous species present in the rainy season. In contrast, common species are reflected in trends of similar foraging patterns, as well as the presence of common species between areas^38^. In addition to different levels of disturbance, differences in floristic composition between areas may also be due to edaphic variation^40^.

Our second hypothesis was refuted because the difference in the richness of exotic species between the areas. Plausible explanations for this finding are that, in general, exotic herbaceous species are commonly used for forage in the semi-arid region of Brazil^41^. Herbaceous species comprise the primary component of the ruminant diet. However, in the midst of their occurrence restricted to the short rainy period, exotic species, mainly of Fabaceae and Poaceae, have been introduced to increase the forage availability, which currently represents an important attribute of forage resources in the Caatinga^41–43^. At the same time, and to also increase the availability of forage resources, the cultivation of species by agro-pastoralists may be common in their properties^44^, mainly exotics, such as *Prosopis juliflora*, that have high adaptive potential and governmental incentives^45^.

Regarding use patterns, according to the data presented here it is possible to state that agro-pastoralists ’ experiences with herding activities provide an accumulation of a vast knowledge about forage resources^15^. This knowledge allows forage resources to be characterized by their potential according to a variety of criteria associated with seasonal variation and qualitative attributes, as commonly found by other studies^14–17,37^. Such criteria are often revealed by qualitative approaches that define the valuation perception of resources. Thus, nutritional value and palatability can be implicitly associated with the definitions of “*É uma ração boa*” (it is a good food), “*o bicho gosta muito*” (the animals like it very much) and “*Rico em proteínas*” (rich in protein).

It should be added that the establishment of intrinsic relationships with resources allows a particular understanding at a high level of detail^15,35^, such as changes in palatability throughout development with descriptions including chemical^17^ and structural changes. Studies confirm that some Caatinga species vary in their chemical composition during leaf maturation, which influences nutritional quality^17,46^.

In addition to revealing the domain of information, this body of knowledge allows maximizing forage use based on nutritional properties weighted by availability^14,37^. Nunes^37^ confirmed that the forage species selected by informants and the criteria they adopted coincided with nutritional values measured by the literature, and that, as also found in the present study, younger plants were recognized as highly appreciated by animals. This appreciation is due to the greater palatability of plant organs at this stage^47^. This is a matter of concern for the sustainability of the Caatinga, since direct or indirect grazing has compromised the regeneration process^12^ since younger individuals are clearly more sensitive to damage^48^.

Also, considering the potential of Caatinga, we suggest that investment through government actions encourage the cultivation of native species to ensure the production of forage and, consequently, guarantee the sustainability of livestock activity and the ecosystem in question.

## Conclusions

Our results point to multiple factors that influence patterns of forage use. Such a set of information has guided management practices and contribute to a better understanding of use of forage resources, and indicate that the inclusion of local ecological knowledge is highly necessary in livestock management measures since local experiences add a diversity of information and particularities. The Caatinga is a source of great potential, but seasonality is an important component in the demarcation of uses throughout the seasons since it influences in the extension of knowledge by agro-pastoralists and the palatability of species for ruminants (Fig. 4). On the other hand, the supply of resources and the floristic diversity can shape on management practices and use profile by agro-pastoralists. Residents of regions with greater vegetation cover know and use a greater number of plant species due to their greater availability. Combined with the vast availability of resources offered by Caatinga, the knowledge of agro-pastoralists regarding ecological aspects, nutritional value and preference, among other characteristics, comprises a key component for its use.

## Supplementary Information

Supplementary Information.
