# Supplementary material for: Vegetation cover and seasonality as indicators for selection of forage resources by local agro-pastoralists in the Brazilian semiarid region
Source: Sci Rep. 2022 Sep 7;12:15174. doi: 10.1038/s41598-022-18282-w (PMC9452531; doi:10.1038/s41598-022-18282-w)
Supplement: Supplementary file 1 — Supplementary Information. [file 41598_2022_18282_MOESM1_ESM.docx]

**Appendix 1**. Forage species cited by respondents in areas with different levels of vegetation cover, Area I and Area II, in the semi-arid region of Brazil. ST: Stratum, BO: Biogeographic origin, H: Herbaceous, W: Woody, N: Native, E: Exotic, LF: Leaf, BR: Branch, BA: Bark, FR: Fruit, FLR: Flower.*species that do not have a voucher number.

| **Family / Species** | **Voucher**  **ID number** | **Popular name** | **Consumed part** | **ES** | **OB** | **Area I** | **Area II** |
| --- | --- | --- | --- | --- | --- | --- | --- |
| **Amaranthaceae** | ***** |  |  |  |  |  |  |
| *Alternanthera brasiliana* (L.) Kuntze | * | Ervanço | FL | H | N | 1 | 1 |
| *Amaranthus viridis* L. | * | Bredo | PI | H | E | - | 7 |
| **Anacardiaceae** | ***** |  |  |  |  |  |  |
| *Myracrodruon urundeuva* Allemão | 1991 | Aroeira | FL, CSC, SE | L | N | 27 | 41 |
| *Schinopsis brasiliensis* Engl. | 2009 | Braúna | FL, CSC | L | N | 16 | 25 |
| *Spondias tuberosa* Arruda | * | Umbuzeiro | FL, CSC, FRU, RA | L | N | 27 | 45 |
| **Apocynaceae** | ***** |  |  |  |  |  |  |
| *Aspidosperma pyrifolium* Mart. & Zucc | 1995 | Pereiro | FL | L | N | 31 | 37 |
| *Calotropis procera* (Aiton) W.T.Aiton | * | Algodão de Seda | FL | L | E | 2 | - |
| **Asteraceae** | ***** |  |  |  |  |  |  |
| *Acanthospermum hispidum* DC. | * | Carrapixo Cigano | FL | H | N | 8 | 2 |
| *Chaptalia nutans* (L.) Pol. | * | Língua de vaca | FL | H | N | 1 | - |
| **Bignoniaceae** | ***** |  |  |  |  |  |  |
| *Tabebuia aurea* (Silva Manso) Benth. & Hook.f. ex S.Moore | * | Caibeira | FL, FLR, CSC | L | N | 22 | 9 |
| *Euploca procumbens* (Mill.) Diane & Hilger | * | Mato azul | PI | H | E | 5 | - |
| **Bromeliácea** | ***** |  |  |  |  |  |  |
| *Bromelia laciniosa* Mart. ex Schult. & Schult. f. | * | Macambira | FL, TB | H | N | 23 | 31 |
| *Neoglaziovia variegata* (Arruda) Mez | * | Caroá | FL, TB | H | N | 1 | 6 |
| **Burseraceae** | ***** |  |  |  |  |  |  |
| *Commiphora leptophloeos* (Mart.) J.B.Gillett | * | Umburana | FL, CSC | L | N | 9 | 30 |
| **Cactaceae** | ***** |  |  |  |  |  |  |
| *Cereus jamacaru* DC. | * | Cardeiro | PI, FR | L | N | 11 | 32 |
| *Melocactus zehntneri* (Britton & Rose) Luetzelb. | * | Coroa de frade | PI | H | N | 4 | 17 |
| *Opuntia ficus-indica* (L.) Mill. | * | Palma | CL | L | E | 1 | 18 |
| *Pilosocereus gounellei* (F.A.C.Weber) Byles & Rowley | * | Xique Xique | CL | L | N | 26 | 36 |
| *Pilosocereus pachycladus* F.Ritter | * | Facheiro | RA, FL, FR | L | N | 15 | 38 |
| *Tacinga palmadora* (Britton & Rose) N.P.Taylor & Stuppy | * | Palmatória | CL, FR | L | N | 16 | 15 |
| **Capparaceae** | ***** |  |  |  |  |  |  |
| *Cynophalla flexuosa* (L.) J.Presl | 2014 | Feijão Bravo | FL, SE | L | N | 19 | 37 |
| **Celastraceae** | ***** |  |  |  |  |  |  |
| *Maytenus rigida* Mart. | * | Bom nome | FL | L | N | - | 5 |
| **Combretaceae** | ***** |  |  |  |  |  |  |
| *Combretum leprosum* Mart. | 1990 | Mufumbo | FL | L | N | 6 | - |
| **Commelinaceae** | ***** |  |  |  |  |  |  |
| *Murdannia nudiflora* (L.) Brenan | * | Erva de Santa Luzia | FL | H | E | - | 1 |
| **Convolvulaceae** | ***** |  |  |  |  |  |  |
| *Ipomoea* L. | * | Jitirana | FL | H | N | 8 | 19 |
| *Jacquemontia tamnifolia* (L.) Griseb. | * | Amarra cachorro | PI | H | N | - | 1 |
| **Cucurbitaceae** | ***** |  |  |  |  |  |  |
| *Momordica charantia* L. | * | Melão são caetano | PI | H | E | 1 | - |
| **Cyperaceae** | ***** |  |  |  |  |  |  |
| *Cyperus compressus* L. | * | Barba de bode | PI | H | N | 2 | 1 |
| **Euphorbiaceae** | ***** |  |  |  |  |  |  |
| *Cnidoscolus quercifolius* Pohl | * | Favela | FL | L | N | - | 5 |
| *Croton blanchetianus* Baill. | * | Marmeleiro | FL, CSC | L | N | 31 | 43 |
| *Jatropha mollissima* (Pohl) Baill. | 1984 | Pinhão | FL | L | N | - | 3 |
| *Manihot glaziovii* Müll.Arg. | * | Maniçoba | FL | L | N | 11 | 43 |
| *Ricinus communis* L. | * | Carrapateira | FL | L | N | 7 | 1 |
| *Sapium glandulosum* (L.) Morong | 2010 | Burra leitera | FL | L | N | - | 6 |
| **Fabaceae** | ***** |  |  |  |  |  |  |
| *Amburana cearensis* (Allemão) A.C.Sm. | 1981 | Cumarú | FL | L | N | - | 1 |
| *Anadenanthera colubrina* (Vell.) Brenan | 1982 | Angico | FL, CSC | L | N | 9 | 29 |
| *Bauhinia cheilantha* (Bong.) Steud. | 1986 | Mororó | FL, CSC | L | N | 4 | 22 |
| *Libidibia ferrea* (Mart. ex Tul.) L.P.Queiroz var. férrea | 1996 | Pau ferro | FL, FR | L | N | 6 | 6 |
| *Cenostigma pyramidale* (Tul.) E. Gagnon & G.P. Lewis | 1988 | Catingueira | FL, FR, CSC | L | N | 35 | 46 |
| *Erythrina velutina* Willd. | * | Mulungu | FL, CSC | L | N | 5 | 8 |
| *Indigofera hirsuta* L. | * | Anil | FL | H | N | 2 | - |
| *Leucaena leucocephala* (Lam.) de Wit | * | Lucena | FL | L | E | 1 | 6 |
| *Lonchocarpus sericeus* (Poir.) Kunth ex DC. | * | Ingazeira | FL | L | N | 1 | - |
| *Mimosa ophthalmocentra* Mart. ex Benth. | 1992 | Jurema de embira | FL, CSC, SE | L | N | 14 | 21 |
| *Mimosa tenuiflora* (Willd.) Poir. | 1989 | Jurema preta | FL, CSC, SE | L | N | 4 | 40 |
| *Piptadenia stipulacea* (Benth.) Ducke | 1979 | Jurema branca | FL | L | N | 2 | 25 |
| *Prosopis juliflora* (Sw.) DC. | * | Algaroba | FR, CSC | L | N | 24 | 15 |
| *Senegalia tenuifolia* (L.) Britton & Rose | * | Unha de gato | FL | L | N | - | 2 |
| *Senna obtusifolia* (L.) H.S.Irwin & Barneby | * | Mata pasto | FL | H | N | - | 3 |
| *Stylosanthes humilis* Kunth | * | Capim de ovelha | FL | H | N | - | 3 |
| **Lamiaceae** | ***** |  |  |  |  |  |  |
| *Mesosphaerum suaveolens* (L.) Kuntze | * | Bamburrá | FL | H | N | - | 3 |
| **Loasaceae** | ***** |  |  |  |  |  |  |
| *Mentzelia aspera* L. | * | Amor de velho | FL | H | N | 1 | 1 |
| **Malvaceae** | ***** |  |  |  |  |  |  |
| *Ceiba glaziovii* (Kuntze) K.Schum. | 1993 | Barriguda | FL | L | N | - | 12 |
| *Herissantia crispa* (L.) Brizicky | * | Mela bode | FL | H | N | - | 1 |
| *Malva sp.* | * | Malva | FL | H | N | 14 | 7 |
| *Melochia sp*. | * | Capa Bode | FL,PI | H | N | 3 | - |
| *Melochia tomentosa* L. | 1980 | Malva roxa | FL | H | E | 4 | 1 |
| *Pseudobombax marginatum* (A.St.-Hil., Juss. & Cambess.) A.Robyns | * | Embiratã | FL | L | N | - | 7 |
| *Sida cordifolia* L. | * | Malva branca | FL | H | N | 3 | 3 |
| *Sida rhombifolia* L. | * | Relógio | FL,PI | H | N | - | 2 |
| **Myrtaceae** | * |  |  |  |  |  |  |
| *Campomanesia eugenioides* (Cambess.) D.Legrand ex Landrum | * | Murta | FL | H | N | - | 1 |
| **Não identificada** | ***** |  |  |  |  |  |  |
|  | * | Amor de velho | FL | - | - | 1 | 1 |
| - | * | Belota | FL | - | - | 1 | - |
| - | * | Cabeça de boi | FL | - | - | 1 | - |
| - | * | Fava de preá | FL | - | - | 1 | - |
| - | * | Favinha do mato | FL | - | - | - | 1 |
| - | * | Cipó de rato | FL | - | - | - | 1 |
| - | * | Cebola braba | FL | - | - | - | 1 |
| - | * | Erva de flor amarela | FL | - | - | 1 | - |
| - | * | Erva de reto | FL | - | - | 1 | - |
| - | * | Plantinhas pequenas | FL | - | - | 3 | - |
| - | * | Jurema Amoroza | FL | - | - | - | 1 |
| - | * | Jurema d' água | FL | - | - | 1 | - |
| - | * | Vamora | FL | - | - | - | 1 |
| - | * | Salsa | PI | - | - | 1 | - |
| - | * | Jurema braba | FL | - | - | - | 3 |
| - | * | Jurema de Veado | FL | - | - | - | 1 |
| - | * | Matapá | FL | - | - | - | 1 |
| - | * | Pega velho | FL | - | - | - | 1 |
| **Nyctaginaceae** | ***** |  |  |  |  |  |  |
| *Boerhavia diffusa* L. | * | Pega pinto | PI, FL | H | E | 2 | 6 |
| *Guapira hirsuta* (Choisy) Lundell | * | João mole | FL | L | N | 1 | 2 |
| **Passifloraceae** | ***** |  |  |  |  |  |  |
| *Passiflora foetida* L. | * | Maracujá do mato | FL | H | N | 1 | - |
| **Plantaginaceae** | ***** |  |  |  |  |  |  |
| *Scoparia dulcis* L. | * | Vassourinha | FL | H | N | - | 2 |
| *Stemodia maritima* L. | * | Meladinha | FL | H | E | 1 | - |
| **Poaceae** | ***** |  |  |  |  |  |  |
| *Cenchrus ciliaris* L | * | Capim bufa | FL | H | E | 6 | 4 |
| *Anthephora hermaphrodita* (L.) Kuntze | * | Capim | FL | H | E | 12 | 14 |
| *Aristida setifolia* Kunth | * | Capim Panasco | FL | H | N | 13 | 4 |
| *Brachiaria decumbens* Stapf | * | Capim braquiária | FL | H | E | 2 | - |
| *Cenchrus echinatus* L. | * | Capim carrapixo | FL | H | E | - | 1 |
| *Cenchrus purpureus* (Schumach.) Morrone | * | Capim elefante | FL | H | E | 2 | 1 |
| *Eleusine coracana* (L.) Gaertn. | * | Pé de galinha | PI,FL | H | E | - | 3 |
| *Enteropogon mollis* (Nees) Clayton | * | Capim do Mato | FL | H | N | 2 | - |
| *Eragrostis ciliaris* (L.) R.Br. | * | Capim mimoso | FL | H | E | 6 | 4 |
| *Gradua sp.* | *** |  | FL | H | N | 1 | - |
| *Sorghum bicolor* (L.) Moench | * | Capim Sorgo | FL | H | E | 3 | 2 |
| **‎Portulacaceae** | ***** |  |  |  |  |  |  |
| *Portulaca oleracea* L. | * | Berduega | PI | L | E | 3 | - |
| **Rhamnaceae** | ***** |  |  |  |  |  |  |
| *Ziziphus joazeiro* Mart. | * | Joazeiro | FL, FR, CSC, RA | L | N | 30 | 43 |
| **Rubiaceae** | ***** |  |  |  |  |  |  |
| *Amorimia septentrionalis* W.R.Anderson | * | Tinguim | FL | H | N | 8 | 7 |
| **Sapotaceae** | ***** |  |  |  |  |  |  |
| *Sideroxylon obtusifolium* (Roem. & Schult.) T.D.Penn. | 1994 | Quixabeira | FL, FR, CSC | L | N | 32 | 38 |
| **Schizaeaceae** | ***** |  |  |  |  |  |  |
| *Lygodium volubile* Sw. | * | Feijão de lambu | FL | H | N | - | 1 |
| **Solanaceae** | ***** |  |  |  |  |  |  |
| *Physalis angulata* L. | * | Camapu | FL | H | E | - | 2 |
| *Solanum agrarium* Sendtn | * | Gogóia | FL | H | N | - | 1 |
| **Verbenaceae** | ***** |  |  |  |  |  |  |
| *Lippia alnifolia* Mart. & Schaue | * | Alecrim do mato | FL | H | N | - | 1 |
| **Violaceae** | ***** |  |  |  |  |  |  |
| *Pombalia calceolaria* (L.) Paula-Souza | * | Papaconha | FL |  | N | - | 1 |
